# First Detection of Hepatitis B Virus Subgenotype A5, and Characterization of Occult Infection and Hepatocellular Carcinoma-Related Mutations in Latin American and African Immigrants in Brazil

**DOI:** 10.3390/ijms25168602

**Published:** 2024-08-07

**Authors:** Thaís Barbosa Ferreira Sant’Anna, Thaynara Lorrane Silva Martins, Megmar Aparecida dos Santos Carneiro, Sheila Araujo Teles, Karlla Antonieta Amorim Caetano, Natalia Motta de Araujo

**Affiliations:** 1 Laboratory of Molecular Virology and Parasitology, Oswaldo Cruz Institute, FIOCRUZ, Rio de Janeiro 21040-900, Brazil; thaisbfsantanna@gmail.com; 2 Faculty of Nursing, Federal University of Goiás, Goiania 74605-080, Brazil; thaynara3@hotmail.com (T.L.S.M.); sheilafen@gmail.com (S.A.T.); karlla@ufg.br (K.A.A.C.); 3 Institute of Tropical Medicine and Public Health, Federal University of Goiás, Goiania 74605-050, Brazil; megmar242@gmail.com

**Keywords:** HBV, molecular epidemiology, genotypes, phylogenetic analysis, immigration

## Abstract

This study aims to characterize the molecular profile of the hepatitis B virus (HBV) among socially vulnerable immigrants residing in Brazil to investigate the introduction of uncommon HBV strains into the country. Serum samples from 102 immigrants with positive serology for the HBV core antibody (anti-HBc) were tested for the presence of HBV DNA by PCR assays. Among these, 24 were also positive for the HBV surface antigen (HBsAg). The full or partial genome was sequenced to determine genotype by phylogenetic analysis. Participants were from Haiti (79.4%), Guinea-Bissau (11.8%), Venezuela (7.8%), and Colombia (1%). Of the 21 HBV DNA-positive samples, subgenotypes A1 (52.4%), A5 (28.6%), E (9.5%), F2 (4.8%), and F3 (4.8%) were identified. Among the 78 HBsAg-negative participants, four were positive for HBV DNA, resulting in an occult HBV infection rate of 5.1%. Phylogenetic analysis suggested that most strains were likely introduced to Brazil by migration. Importantly, 80% of A5 sequences had the A1762T/G1764A double mutation, linked to an increased risk of hepatocellular carcinoma development. In conclusion, this study is the first report of HBV subgenotype A5 in Brazil, shedding new light on the diversity of HBV strains circulating in the country. Understanding the genetic diversity of HBV in immigrant communities can lead to better prevention and control strategies, benefiting both immigrants and wider society.

## 1. Introduction

Hepatitis B is a highly prevalent viral infection in humans and a serious global public health problem. Hepatitis B virus (HBV) has infected one-third of the global population, with an estimated 296 million chronic infections [1]. In 2019, the World Health Organization estimated that hepatitis B was linked to approximately 820,000 deaths, primarily associated with cirrhosis and hepatocellular carcinoma (HCC). Despite the availability of a prophylactic vaccine since the 1980s, around 1.5 million new infections are reported annually [1]. The spread of chronic HBV infection varies greatly by geographical location. In regions with a high prevalence of chronic carriers, HBV infection is frequently transmitted at birth or during early childhood, while in areas with a low prevalence, it is typically acquired during adulthood through percutaneous or sexual transmission [2]. HBV infection acquired in adulthood results in chronic hepatitis in fewer than 5% of cases, whereas infection in infancy and early childhood causes chronicity in about 95% of cases. HBV surface antigen (HBsAg) is the established serological marker for the diagnosis of acute or chronic HBV infection. However, the presence of HBV DNA in the blood and/or liver of individuals who test negative for HBsAg is indicative of a unique form of chronic viral infection known as occult HBV infection (OBI). A low HBV replication rate due to the host’s immune response may contribute to OBI in the majority of cases. Mutations altering the antigenicity of HBsAg, known as immune escape mutations, can also result in occult infection, impeding detection by commercial assays [3].

HBV is the prototype member of the family *Hepadnaviridae*, containing a partially double-stranded relaxed circular DNA genome approximately 3200 nucleotides (nt) in length [4,5]. This family is characterized by a distinctive viral replication cycle including the activity of an error-prone reverse transcriptase, resulting in the formation of significant variability across HBV isolates [6,7]. HBV has been classified into nine genotypes (A–I), based on a greater than 7.5% genomic sequence divergence, with a potential genotype (J) recovered from a single individual. In addition, due to the large intra-genotypic diversity, genotypes A–D, F, and I have been further divided into subgenotypes based on 4% to 7.5% nucleotide divergence in the complete genome [8]. Frequencies of clinically relevant mutations (i.e., immune escape-, drug resistance-, and HCC-associated mutations) vary significantly between HBV genotypes [9], while differences in transmission routes, disease progression, response to antiviral therapies, and clinical outcome have also been demonstrated [10,11,12]. In particular, patients infected with genotype C have more severe liver disease, including cirrhosis and HCC, compared to those with genotype B [13,14]. Additionally, genotype D is associated with more progressive liver disease compared to genotype A [15,16].

Recent findings of HBV DNA in archeological remains demonstrate that humans have been infected with HBV for millennia [17]. Given that HBV lacks environmental or animal reservoirs, its spread is closely associated with human dispersion. The global distribution of HBV genotypes and, occasionally, subgenotypes displays a significant geographic association, which most likely reflects historical human migration patterns [18,19,20]. Genotype A is frequently found in Africa (subgenotypes A1, A3, and A4), the Caribbean (A1 and A5), Europe and North America (A2), and South America (A1 and A2), while genotypes B and C are found essentially in Asia. Genotype E is limited to West and Central Africa, and genotypes F and H are found in Amerindian populations. Genotypes D and G have a ubiquitous geographic distribution, while the more recent genotypes I and J were described in China, India, Laos, and Vietnam (I), and Japan (J) [21,22].

The 2016 World Health Assembly endorsed the Global Health Sector Strategy including a goal to eliminate viral hepatitis as a public health threat by 2030, reducing new infections by 90% and mortality by 65% [23]. However, in order to meet these objectives, it is critical to reach socially vulnerable subpopulations at risk of acquiring or transmitting HBV infection, such as immigrants and refugees, a population that is growing globally. The molecular characteristics of HBV infection in immigrants reflect those of their home countries. The flow of immigrants may contribute to changes in the frequencies of HBV (sub)genotypes and the introduction of uncommon strains in host countries [24,25].

Brazil is the largest country in the Southern Hemisphere and traditionally a destination country for different migratory flows that have occurred throughout history. Despite not receiving significant immigration numbers today, Brazil is experiencing a gradual growth in migratory movements. In 2021, over 150,000 immigrants entered the country, most of them coming from geographical areas with intermediate or high HBV endemicity and living in conditions of poverty and social vulnerability [26]. In Brazil, HBV genotypes A, D, and F are mainly found countrywide, reflecting the contribution of the African (A1), European (A2 and D), and Amerindian (F) people in the formation of the Brazilian population [27,28,29]. Data on the molecular characterization of HBV in immigrant populations in Brazil are extremely scarce. Therefore, this study investigated the HBV (sub)genotypes, and the occurrence of OBI and clinically relevant viral mutations among socially vulnerable immigrants from Latin America and Africa residing in Brazil, to determine the potential introduction of uncommon HBV strains into the country.

## 2. Results

Between July 2019 and January 2020, socially vulnerable immigrants and refugees residing in Goiás, Central-West Brazil, were recruited for a study investigating the seroprevalence of hepatitis B and C infections and their associated epidemiological factors [30]. Of the original 365 participants from the previous study, 102 individuals tested positive for the HBV core antibody (anti-HBc), a serological marker of exposure to HBV, and were included in the present investigation. From these, 24 (23.5%) were also positive for HBsAg. Participants self-declared the following countries of origin: Haiti (n = 81; 79.4%), Guinea-Bissau (n = 12; 11.8%), Venezuela (n = 8; 7.8%), and Colombia (n = 1; 1%). Most of them were male (68.6%) and aged 31–50 years (56.9%). HBV DNA was detected in 21/102 (20.6%) individuals (Table 1). Among the 78 HBsAg-negative subjects, four were positive for HBV DNA, resulting in an OBI rate of 5.1%.

**Figure 1:**
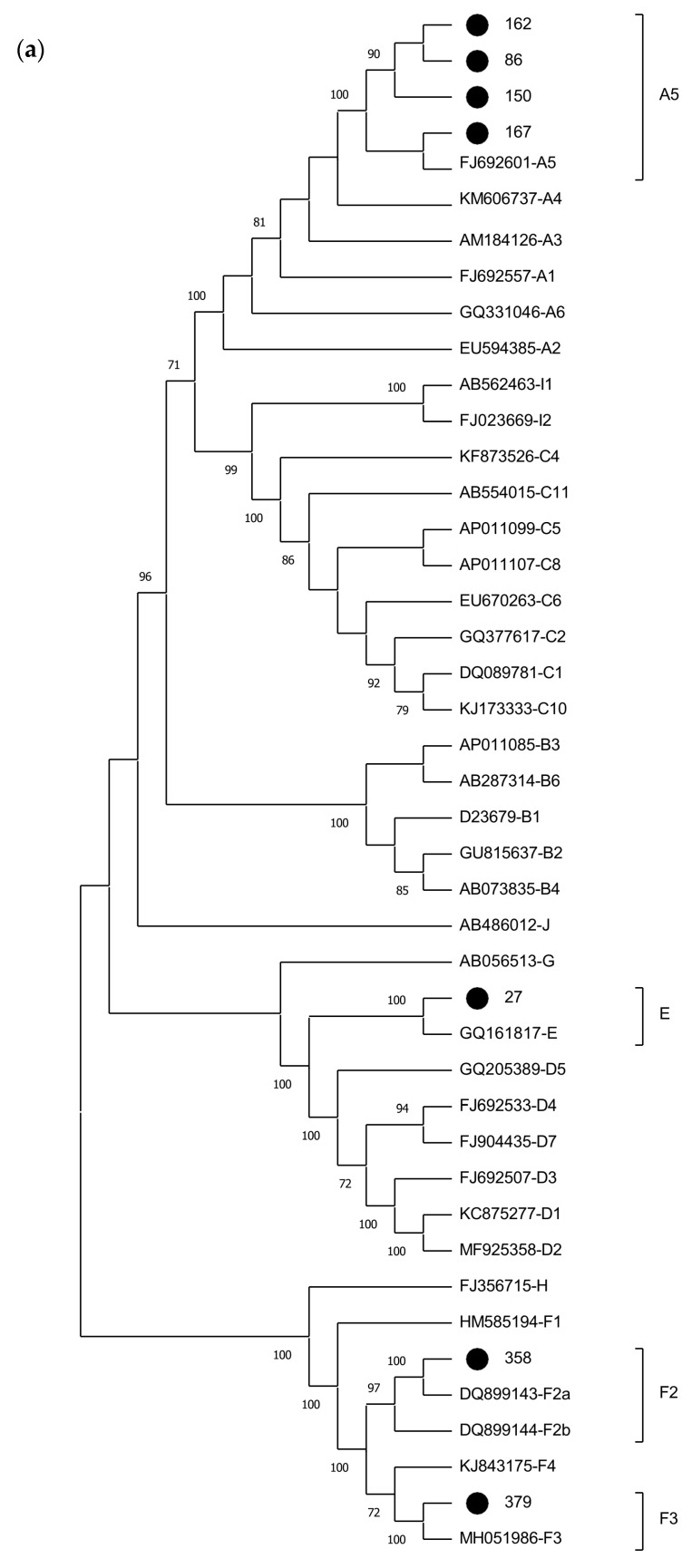

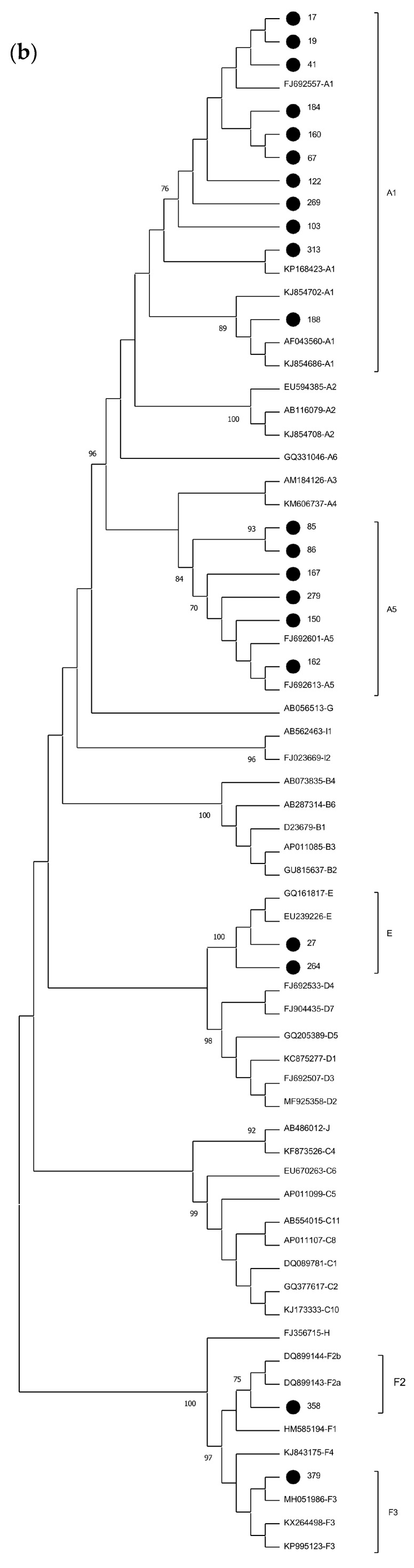

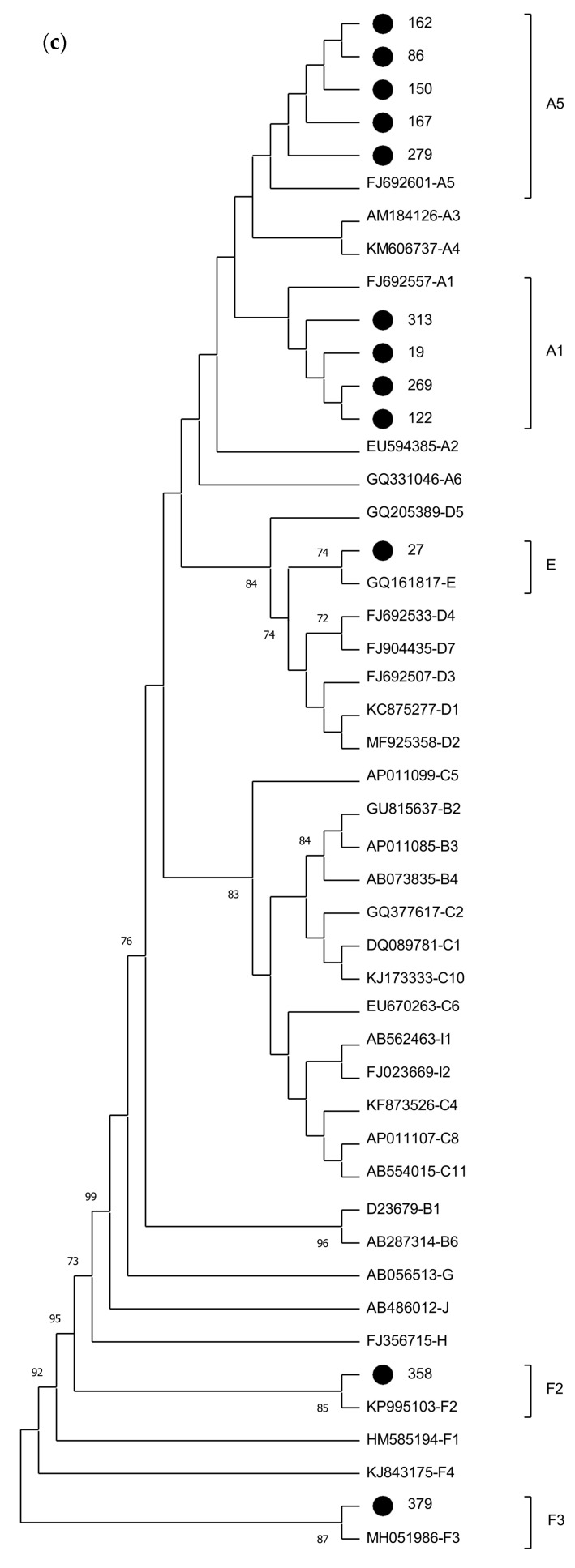
Phylogenetic analysis of HBV sequences inferred using the Maximum Likelihood method. Values at internal nodes indicate the percentage of 1000 bootstrap replicates that support the group (values below 70 were hidden). HBV reference sequences are indicated by their accession numbers followed by their genotype/subgenotype. The sequences generated in this study are identified with a black dot. Phylogenetic tree displayed without scale due to logarithmic scaling option (‘Toggle scaling of the tree’) in MEGA 11 software. (**a**) Analysis of the full-length sequences. (**b**) Analysis of partial S gene region (581 bp). (**c**) Analysis of partial X and pre-C/C gene regions (428 pb).

Phylogenetic analysis of the 21 samples positive for HBV DNA demonstrated the presence of subgenotypes A1 (n = 11; 52.4%), A5 (n = 6; 28.6%), E (n = 2; 9.5%), F2 (n = 1; 4.8%), and F3 (n = 1; 4.8%). In addition to amplifying the S and pre-C/C regions, complete genome amplification was specifically performed for samples classified as genotypes A5, E, F2, and F3, which are rare in Brazil. Phylogenetic trees representing the complete genome (n = 7), S (n = 21), and X/pre-C/C (n = 12) sequences obtained in this study are shown in Figure 1. Sample classification into genotypes and subgenotypes remained unchanged in all three phylogenetic analyses (Figure 1).

Individuals infected with HBV subgenotype A1 were immigrants from Haiti (n = 10) and Guinea-Bissau (n = 1), while those infected with subgenotype A5 were exclusively from Haiti. The two cases of genotype E infection were from Haiti and Guinea-Bissau, respectively. Subgenotypes F2 and F3 were both found in Venezuelan immigrants. The four cases of OBI corresponded to genotypes A1 (n = 3) and A5 (n = 1) and were detected in immigrants from Guinea-Bissau (A1) and Haiti (A1 and A5) (Table 2).

**Figure 2:**
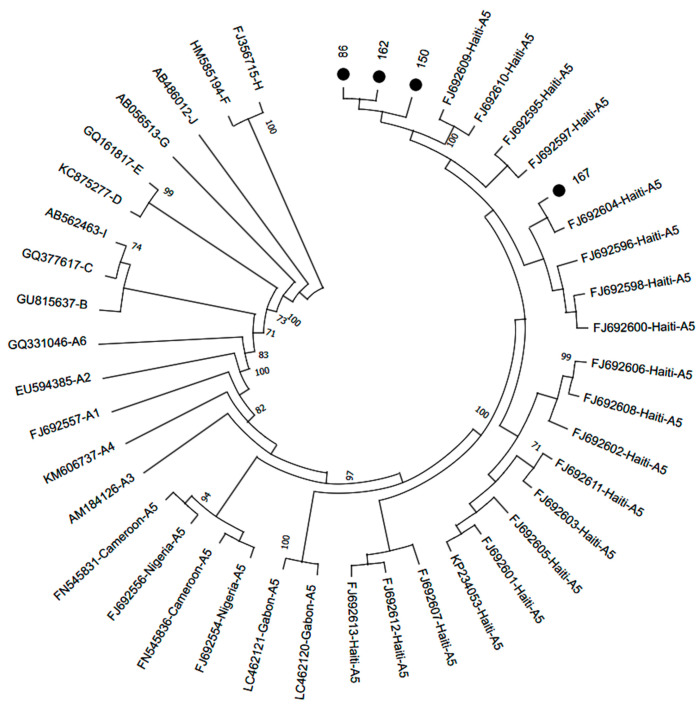
Phylogenetic analysis of HBV complete genome sequences inferred using the Maximum Likelihood method. Phylogenetic tree of 29 subgenotype A5 sequences (four sequences recovered in this study and identified with a black dot, and 25 worldwide sequences identified by their accession number and country of origin, obtained from a dataset previously published [9] and subsequently updated). HBV reference sequences for the other HBV (sub)genotypes are indicated by their accession numbers followed by their genotype/subgenotype. Values at internal nodes indicate the percentage of 1000 bootstrap replicates that support the group (values below 70 were hidden). Phylogenetic tree displayed without scale due to logarithmic scaling option (‘Toggle scaling of the tree’) in MEGA 11 software.

Considering the importance of detecting subgenotype A5 circulation in Brazil, an additional phylogenetic analysis was performed on the complete HBV genomes classified as subgenotype A5 from this study. This analysis included 25 full-length sequences of the same subgenotype available in GenBank to enhance the robustness of the classification and to analyze the clustering of these sequences with other A5 sequences from different worldwide locations. All four A5 complete genomes obtained from Haitian participants clustered with other sequences from Haiti, forming a distinct cluster (bootstrap support of 100%) separate from the sequences from the African continent, including those from Cameroon, Gabon, and Nigeria (Figure 2).

**Figure 3:**
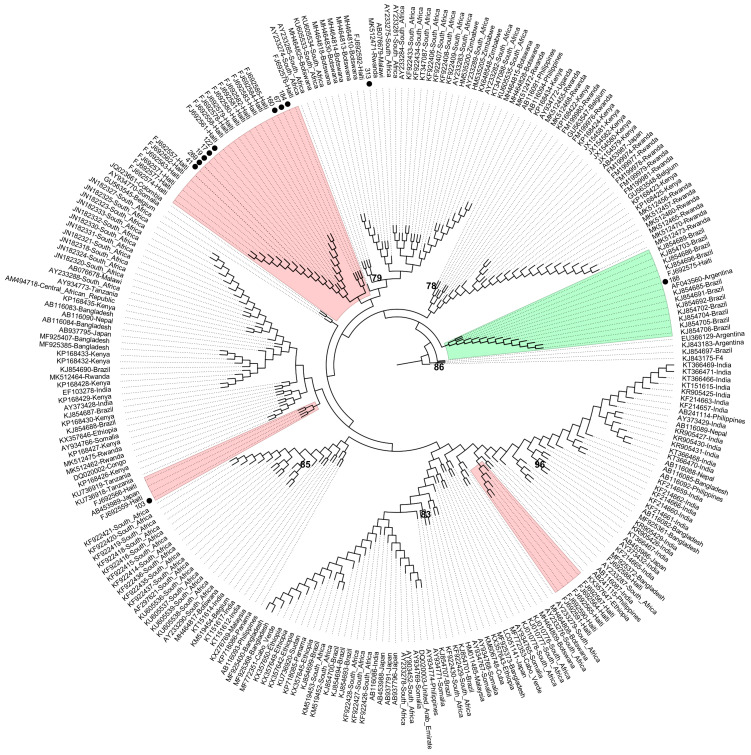
Phylogenetic analysis of HBV partial S gene region (581 bp) inferred using the Maximum Likelihood method. Phylogenetic tree of 270 subgenotype A1 sequences (11 sequences recovered in this study and identified with a black dot, and 259 worldwide sequences identified by their accession number and country of origin, obtained from a dataset previously published [9] and subsequently updated). The sequence with GenBank accession number KJ843175 (subgenotype F4) was used as an outgroup. The sequence clusters from Brazil and Haiti are highlighted in green and pink, respectively. Values at internal nodes indicate the percentage of 1000 bootstrap replicates that support the group (values below 70 were hidden). Phylogenetic tree displayed without scale due to logarithmic scaling option (‘Toggle scaling of the tree’) in MEGA 11 software.

**Figure 4:**
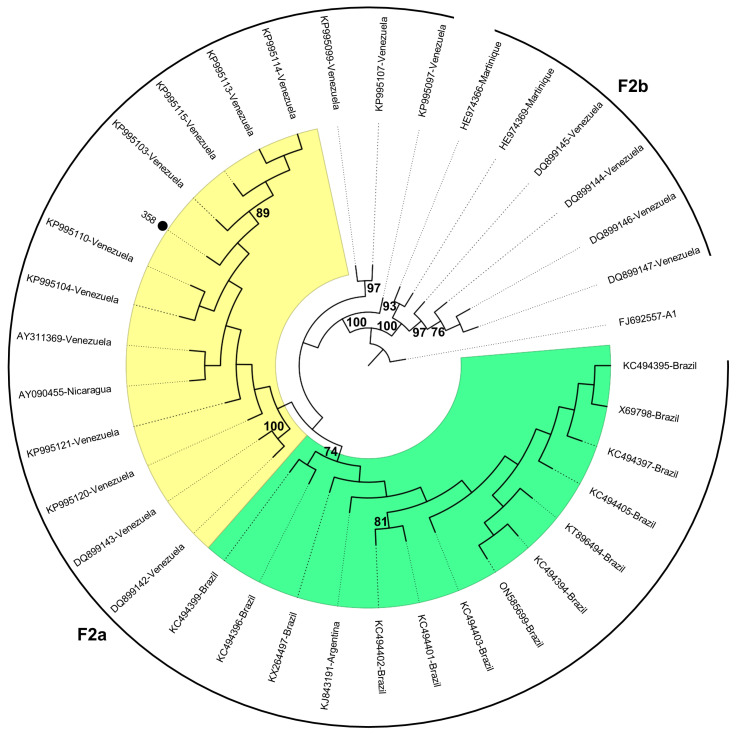
Phylogenetic analysis of HBV complete genome sequences inferred using the Maximum Likelihood method. Phylogenetic tree of 36 subgenotype F2 sequences (one sequence recovered in this study and identified with a black dot, and 35 worldwide sequences identified by their accession number and country of origin, obtained from a dataset previously published [9] and subsequently updated). The sequence with GenBank accession number FJ692557 (subgenotype A1) was used as an outgroup. The sequence clusters from Brazil and Venezuela are highlighted in green and yellow, respectively. Values at internal nodes indicate the percentage of 1000 bootstrap replicates that support the group (values below 70 were hidden). Phylogenetic tree displayed without scale due to logarithmic scaling option (‘Toggle scaling of the tree’) in MEGA 11 software.

Given that subgenotypes A1 and F2 are commonly found in Brazil, additional phylogenetic analyses were conducted using 270 S gene sequences (A1) and 36 full-length sequences (F2) to gain potential insights into whether the individuals may have brought these strains to Brazil or became infected after their arrival. Among the 10 sequences of subgenotype A1 obtained from the Haitian participants, eight (80%) grouped into clusters composed almost exclusively of sequences from Haiti, whereas the remaining two clustered with Brazilian (ID 188) and African (ID 313) sequences, respectively. The single A1 sequence obtained from the immigrant from Guinea-Bissau (ID 67) grouped in the cluster of sequences from Haiti (Figure 3). The subgenotype F2 complete genome retrieved from a Venezuelan participant (ID 358) grouped with Venezuelan sequences, more specifically into the F2a clade (Figure 4).

Regarding the presence of mutations of clinical relevance, none of the sequences showed immune escape- or drug resistance-associated mutations. Nonetheless, mutations linked to HCC were identified, including the A1762T/G1764A mutation in 58.3% (7/12) of pre-C/C sequences and the G1896A stop-codon mutation in 16.7% (2/12) of these sequences. Remarkably, four out of five (80%) subgenotype A5 sequences were positive for A1762T/G1764A. Additionally, 12-nucleotide insertions in the S region were found in two sequences (genotypes A1 and E), while a 21-nucleotide deletion was detected in the pre-S2 region of the F2 subgenotype sequence (Table 2).

## 3. Discussion

To our knowledge, this is the first study on the molecular characterization of HBV in Latin American and African immigrants residing in Brazil. Furthermore, this study demonstrates for the first time the circulation of the rare HBV subgenotype A5 in the country. This subgenotype exhibits a distinct distribution pattern, including West and Central African countries, and Haiti, a country on the island of Hispaniola in the Greater Antilles archipelago of the Caribbean Sea. HBV A5 strains are thought to have spread during the slave trade from African nations to Haiti, where >90% of the population descended from African slaves [31]. The main causes of the recent migratory flows from Haiti have been economic hardship, exacerbated by natural disasters such as the earthquake in 2010 and Hurricane Matthew in 2016 [32]. The migration crisis in Haiti is particularly relevant to Brazil’s migration policies. The present study confirms the predominance of this nationality, with 79.4% of participants being Haitians. HBV (sub)genotypes A1, A5, and E were found in 58.8%, 35.3%, and 5.9% of them, respectively. These findings are consistent with those of Andernach and colleagues (2009) [31], who reported that among pregnant Haitian women surveyed, A1, A5, and E were found in 43%, 19.6%, and 6.1%, respectively. The introduction of HBV A5 strains from Haiti to Brazil is plausible, as this subgenotype had not previously been detected in the country. On the other hand, since subgenotype A1 is the most prevalent subgenotype in Brazil [27], an expanded phylogenetic analysis with 270 sequences of this subgenotype from different locations worldwide was conducted to explore the potential origin of these strains. Interestingly, 80% of A1 sequences obtained from Haitian participants clustered within Haitian sequences, which may suggest an introduction of these A1 strains from Haiti to Brazil.

The discovery of two HBV genotype E strains among immigrants from Haiti and Guinea-Bissau is also of epidemiological relevance, considering that this genotype is extremely rare in Brazil, with a few reports of detection mainly in individuals with connections to the African continent [33,34]. Differing from Brazil and Haiti, HBV genotype E is primarily found in Guinea-Bissau, a country on the western coast of Africa [31,35]. Despite being the most prevalent genotype in West Africa, genotype E has been understudied [36]. Notably, the low prevalence of this genotype in the African descendant populations in the Americas suggests that genotype E was not circulating during the slave trade period (16th to 19th centuries). Furthermore, the low genetic diversity, phylogeographic analyses, and evidence of genotype E in ancient HBV samples point to a relatively recent reintroduction of this genotype into the human population [31,36].

HBV genotype F is assumed to represent the original genotype of the aboriginal populations of the Americas, since it has been detected in high frequencies in various South and Central American countries [37], as well as in native Alaskan communities [38]. Six subgenotypes have been described for HBV genotype F (F1–F6), and different clades have been proposed for subgenotype F1 (F1a, b, c, and d) and F2 (F2a and b) [39]. Unlike other Latin American countries, Brazil has a low prevalence of genotype F, even in the northern region, which includes the Amazon basin, where the indigenous Amerindian population is predominant [28]. Despite its low prevalence, genotype F strains are widespread throughout the country, with F2a being the predominant F subgenotype and the original native HBV of the Brazilian population [40]. In this study, subgenotypes F2a and F3 were identified in immigrants from Venezuela, where these subgenotypes are common [37]. The complete F2 genome sequence grouped within the F2a Venezuelan monophyletic cluster, suggesting that the individual might have acquired the infection in their home country. Similarly, the F3 strain was likely introduced to Brazil by the Venezuelan immigrant, as reports of subgenotype F3 in the country are scarce [41].

Mutations in the basal core promoter region (A1762T/G1764A) and a stop-codon mutation in the pre-C region (G1896A), which decrease/abolish HBeAg expression while enhancing viral replication, as well as deletions in the pre-S region, are associated with an elevated risk of HCC [42]. Importantly, A1762T/G1764A was present at much higher frequencies in A1 and A5 subgenotype sequences (75% and 80%, respectively) obtained in this study, compared to previous observations (26.4% and 31.3%, respectively) [9]. These results draw attention to the introduction in Brazil of HBV strains with greater oncogenic potential. In Brazil, 10.598 deaths were attributed to HCC in 2021 [43], with chronic HBV infection accounting for 22.2% of the cases [44].

OBI raises concerns among researchers worldwide, as little is known about the clinical impact of this infection and its role in the transmission of the virus. Among the immigrant population studied, OBI was detected in three individuals infected with subgenotype A1 and in one infected with A5, yielding an OBI prevalence of 5.1% (n = 4/78) in this group. Numerous studies have shown that OBI rates vary substantially across different populations, due to a combination of factors such as the endemicity of HBV infection, the sensitivity of HBV DNA assays, and the biological sample analyzed [3]. Among Brazilian blood donors, OBI was detected in six out of 976 samples (0.6%) that were positive only for anti-HBc [45]. Previous studies conducted in Central Brazil have shown OBI rates of 0% (n = 0/522) in men who have sex with men [46], 3.8% (n = 19/505) in treatment-naïve HIV-infected patients [47], and 12.7% (n = 19/149) in injecting drug users [48].

This study has some limitations that should be acknowledged. First, the analysis was conducted on a limited number of sequences, potentially constraining the depth of insight into the epidemiological context of this population. Second, owing to limits in resources and time, this study exclusively enrolled individuals who tested positive for anti-HBc. The incorporation of seronegative individuals could offer a broader understanding of the prevalence of OBI within this population. Third, due to the limited quantity of available HBV DNA, some samples were only genotyped through sequencing of the S region (seven out of 11, subgenotype A1; and one out of six, subgenotype A5), which could be misleading for differentiating subgenotypes. Additionally, the presence of HBV strains with recombination between different (sub)genotypes may have been overlooked in these samples. Fourth, conducting Bayesian phylogeographic analyses would enable the reconstruction of the spatial and temporal diversification of the rare genotypes A5 and E in Brazil.

In conclusion, this study underscores the importance of investigating HBV (sub)genotypes and mutations in immigrant populations. Our results suggest that the rare HBV subgenotype A5 may have been introduced into Brazil, potentially through Haitian immigrants, but it is also possible that this subgenotype could have arrived via other routes, such as international travel, the use of blood products from different countries, or other migration patterns. The introduction of uncommon (sub)genotypes and variants carrying clinically relevant mutations presents a potential challenge to countries of destination. However, it is important to approach this issue with sensitivity and respect for the experiences and perspectives of immigrant communities. A better understanding of the genetic diversity of HBV in these populations could guide focused epidemiological studies and preventive measures, potentially identifying specific viral strains and transmission patterns, thereby benefiting both immigrants and the broader population. Furthermore, this research can contribute to the global effort to combat HBV, as sharing research findings across regions can lead to more targeted and efficient interventions.

## 4. Materials and Methods

### 4.1. Study Population

This cross-sectional study focused on socially vulnerable immigrants and refugees living in Goiás, Central-West Brazil. From July 2019 to January 2020, participants were recruited for a study examining the seroprevalence of hepatitis B and C infections and their associated epidemiological factors [30]. Out of the initial 365 participants from the previous study, 102 individuals with positive serology for anti-HBc were enrolled in this study. Informed written consent was obtained at the time of sampling from all participants. The Ethics Committee of Federal University of Goiás approved the study protocol (3.243.845 CAAE 06871019.7.0000.5083).

### 4.2. Viral DNA Extraction and PCR Amplification

A total of 10 mL of blood was collected from each participant via peripheral venipuncture in the upper arm. The samples were transferred into test tubes, labeled with participant numbers, placed in an air-conditioned thermal box, and sent to the Multi-user Clinical Research Laboratory (LAMPEC) of the Faculty of Nursing, UFG. The blood samples were then centrifuged to obtain serum, aliquoted, and stored in a −20 °C freezer. In January 2022, an aliquot of serum was sent on dry ice to the Laboratory of Molecular Virology and Parasitology at FIOCRUZ for molecular biology assays. Viral nucleic acids were extracted from 0.2 mL serum using a High Pure Viral Nucleic Acid kit (Roche Diagnostics, Indianapolis, IN, USA) according to the manufacturer’s instructions. Serum samples were tested for the presence of HBV DNA by qualitative polymerase chain reaction (PCR) assays. Negative controls were added in both extraction and PCR procedures. HBV partial S region (581 base pairs, bp) was amplified by semi-nested PCR. The first round of amplification was performed with 2 µL of DNA, 1U of Platinum Taq DNA Polymerase (Invitrogen, Waltham, MA, USA), and sense PS1 (5′-CCATATTCTTGGGAACAAGA-3′, nts 2826–2845) and antisense SR (5′-CGAACCACTGAACAAATGGC-3′, nts 704–685) oligonucleotide primers. Second round PCR was performed with 2 µL of the first round product, 1U of Platinum Taq DNA Polymerase (Invitrogen), sense S1 (5′-CTTCTCGAGGACTGGGGACC-3, nts 124–143), and antisense SR primers. PCR conditions were as follows: 94 °C for 2 min, followed by 35 cycles of 94 °C for 30 s, 52 °C (first step) or 55 °C (nested PCR) for 30 s and 72 °C for 1 min 30 s, and a final extension step at 72 °C for 7 min.

HBV partial X and pre-C/C regions (428 bp) were amplified in a nested PCR assay carried out with 4 µL of DNA, 1U of Platinum Taq DNA Polymerase (Invitrogen), and sense X1 (5′-ACCTCCTTTCCATGGCTGCT-3′, nts 1363–1382) and antisense C2 (5′-CTAACATTGAGATTCCCGAGATTGAGA-3′, nts 2458–2432) oligonucleotide primers. Second round PCR was performed with 4 µL of the first round product, 1U of Platinum Taq DNA Polymerase (Invitrogen), sense X4 (5′-AAGGTCTTACATAAGAGGAC-3, nts 1644–1663) and antisense C3 (5′-TTGCCTGAGTGCAGTATGGT-3′, nts 2071–2052) primers. PCR conditions for both rounds were 94 °C for 2 min, 35 cycles at 94 °C for 30 s, 52 °C for 30 s, and 72 °C for 1 min 30 s, followed by a final elongation at 72 °C for 7 min.

Almost the entire HBV genome (3164 bp) was amplified by nested PCR. The first round was attempted with a protocol modified from Günther and colleagues (1995) [49] using 2 μL of DNA, primers P1-mod (5′-TTTTTCACCTCTGCCTAATCA-3′, nts 1821–1841) and P2-mod (5′-AAAAAGTTGCATGGTGCTGG-3′, nts 1825–1806), and the following PCR profile: denaturation at 94 °C for 4 min, followed by 10 cycles at 94 °C for 40 s, 55 °C for 1 min, and 72 °C for 3 min; 10 cycles of 94 °C for 40 s, 60 °C for 1 min, and 72 °C for 5 min; 10 cycles of 94 °C for 40 s, 62 °C for 1 min, and 72 °C for 7 min; 10 cycles of 94 °C for 40 s, 62 °C for 1 min, and 72 °C for 9 min; and a final extension step at 72 °C for 10 min. Second round PCR was performed with 5 µL of the first round product, and sense P1-nested (5′-ATGTCCTACTGTTCAAGCCTCC-3′, nts 1852–1873) and antisense P2-nested (5′-ATTTATGCCTACAGCCTCCT-3′, nts 1794–1775) primers. PCR conditions were as follows: 94 °C for 4 min, 30 cycles at 94 °C for 30 s, 50 °C for 30 s, and 72 °C for 4 min, followed by a final elongation at 72 °C for 10 min. Both PCR assays were performed using 1U of Platinum Taq DNA polymerase and supplied reagents (Invitrogen) in accordance with product instructions.

### 4.3. Nucleotide Sequencing

Samples with a positive result in the PCR assays were subsequently subjected to nucleotide sequencing. PCR products were purified using the QIAquick PCR Purification kit (QIAGEN, Hilden, Germany). HBV sequences were determined via direct sequencing using a BigDye Terminator Kit v3.1 (Applied Biosystems, Waltham, MA, USA), and sequencing reactions analyzed on an ABI3730xl automated sequencer (Applied Biosystems). Nucleotide sequences obtained during this study were deposited in the GenBank database under accession numbers PP093835–PP093848, PP101105–PP101109, and PP116453–PP116459.

### 4.4. HBV Genotyping and Mutational Analysis

Multiple sequence alignments of the amplified genomic regions were performed using the MUSCLE program implemented in MEGA 11 software, with HBV reference sequences for genotypes A to J [50], and subsequently subjected to Maximum Likelihood phylogenetic analysis for genotyping. The phylogenetic trees were inferred with MEGA 11 software under the best nucleotide substitution model selected from Maximum Likelihood fits of 24 different nucleotide substitution models. The visualization of the phylogenetic trees was performed using the MEGA 11 and FigTree v1.4.4 software programs (http://tree.bio.ed.ac.uk/software/figtree/, accessed on 1 December 2023). For mutational analysis, the presence of 21 clinically relevant positions according to previous reports [9] were examined, including 10 amino acid sites within HBsAg (120, 126, 129, 130, 133, 141, 142, 143, 144, and 145) related to immune escape, three nucleotide positions within the core (C) promoter gene (1753, 1762, and 1764), the pre-C stop-codon mutation G1896A, and pre-S deletions related to HCC development, and seven amino acid positions within the reverse transcriptase (RT) domain of HBV polymerase (180, 181, 184, 202, 204, 236, and 250) related to antiviral resistance.

## Figures and Tables

**Table 1:** Sociodemographic and virological characteristics of 102 anti-HBc positive immigrants residing in Brazil.

Variables	n = 102	(%)
Sex		
Male	70	68.6
Female	32	31.4
Age		
2–11	0	0
12–17	0	0
18–30	38	37.3
31–50	58	56.9
≥51	6	5.9
Country of Origin		
Colombia	1	1
Guinea-Bissau	12	11.8
Haiti	81	79.4
Venezuela	8	7.8
Continent of Origin		
Africa	12	11.8
Caribbean	81	79.4
South America	9	8.8
HBsAg		
Positive	24	23.5
Negative	78	76.5
HBV DNA		
Positive	21	20.6
Negative	81	79.4

**Table 2:** Molecular characteristics of the HBV DNA positive samples from this study.

ID	Country of Origin	HBV Serology		Amplified Genomic Region	Mutation Sites				Subgenotype
		HBsAg	Anti-HBc		HBsAg ^b^	pre-C/C ^c^	RT ^d^	Other	
17	Haiti	Pos	Pos	S	None	NS	None ^e^		A1
19	Haiti	Neg ^a^	Pos	S, pre-C/C	None	A1762T/G1764A	None ^e^		A1
41	Haiti	Pos	Pos	S	None	NS	None ^e^		A1
67	Guinea-Bissau	Neg ^a^	Pos	S	None	NS	None ^e^		A1
103	Haiti	Pos	Pos	S	None	NS	None ^e^	12-nt insertion at S region	A1
122	Haiti	Pos	Pos	S, pre-C/C	None	None	None ^e^		A1
160	Haiti	Pos	Pos	S	None	NS	None ^e^		A1
184	Haiti	Pos	Pos	S	None	NS	None ^e^		A1
188	Haiti	Neg ^a^	Pos	S	None	NS	None ^e^		A1
269	Haiti	Pos	Pos	S, pre-C/C	None	A1762T/G1764A	None ^e^		A1
313	Haiti	Pos	Pos	S, pre-C/C	None	A1762T/G1764A	None ^e^		A1
85	Haiti	Neg^a^	Pos	S	None	NS	None ^e^		A5
86	Haiti	Pos	Pos	complete genome	None	A1762T/G1764A, G1896A	None		A5
150	Haiti	Pos	Pos	complete genome	None	A1762T/G1764A	None		A5
162	Haiti	Pos	Pos	complete genome	None	A1762T/G1764A	None		A5
167	Haiti	Pos	Pos	complete genome	None	A1762T/G1764A	None		A5
279	Haiti	Pos	Pos	S, pre-C/C	None	None	None ^e^		A5
27	Haiti	Pos	Pos	complete genome	None	G1896A	None		E
264	Guinea-Bissau	Pos	Pos	S	None	NS	None ^e^	12-nt insertion at S region	E
358	Venezuela	Pos	Pos	complete genome	None	None	None	21-nt deletion at pre-S2 region	F2
379	Venezuela	Pos	Pos	complete genome	None	None	None		F3

^a^ Occult HBV infection (OBI). ^b^ HBsAg amino acid mutations related to immune escape: P120L/Q/S/T, I/T126A/N/S, Q129H/N/R, G130N/R, M133I/L/T, K141E/I, P142L/S, S/T143L, D144A/E/G, G145R. ^c^ Pre-C/C nucleotide mutations related to HCC development: T1753V, A1762T/G1764A, G1896A. ^d^ Reverse transcriptase (RT) nucleotide mutations related to antiviral resistance: L180M, A181T/V, T184G/S, S202G/I, M204I/V, N236T, M250V. ^e^ Only RT nucleotide mutations L180M, A181T/V, T184G/S were analyzed. NS, not sequenced.

## Data Availability

Nucleotide sequences obtained during this study were deposited in the GenBank database under accession numbers PP093835–PP093848, PP101105–PP101109, and PP116453–PP116459.
